# Digital psychiatry: concepts, framework, and implications

**DOI:** 10.3389/fpsyt.2025.1572444

**Published:** 2025-07-04

**Authors:** Hairong Wu, Ming D. Li

**Affiliations:** State Key Laboratory for Diagnosis and Treatment of Infectious Diseases, The First Affiliated Hospital, Collaborative Innovation Center for Diagnosis and Treatment of Infectious Diseases, Zhejiang University School of Medicine, Hangzhou, China

**Keywords:** digital health, psychiatry, mental health, virtual reality, LLMS, ChatGPT, AGI, GMAI

## Abstract

In this review, we consider digital psychiatry as a specialty to focus on combining the psychiatric clinical practices, psychiatric knowledge, and modern intelligent/digital approaches to automate the psychiatric clinical processes, such as diagnosis and treatment, in order to yield faster, better and consistent results, which is far beyond the development of smartphone apps, virtual reality (VR), and chatbots. Based on the recent advances in large-scale pre-trained models (PTMs), digital humans, VR and other immersive techniques, we here propose a framework to fully-automate the processes of mental health practices, and thus pave the way for digital psychiatric clinics. Specifically, in this paper, we first provide an outline of the related technical developments to digital psychiatry, by describing how digital entity, medical domain knowledge, autonomous agents and VR present new opportunities for practical clinical uses. Second, we introduce some basic mental health related issues in clinical settings, which should be considered in digital psychiatry systems. Third, we propose an outline of developing a fully-automated digital psychiatric system building on the existing artificial intelligence (AI) related technologies. Fourth, we discuss the challenges of implementing the digital psychiatry in the real-world environments. Finally, we discuss some key issues related to patients, medical providers and regulations which could not be avoided to implement the digital psychiatric systems and their applications.

## 1 Introduction

The rapid development of digital and artificial intelligence (AI) techniques provides a feasible solution towards transforming the traditional mental health care into digital psychiatry and mitigating the dilemma of the needs for automating clinical practices and the delivery capabilities. While the applications of these techniques in mental health care were initiated slower than other medical domains, Chat Generative Pre-Trained Transformer (ChatGPT)-like technologies have triggered a significant change of the interest and utilization of digital health in psychiatry research and treatment (1, 2). This increasing prevalence is related not only to digital and AI technologies’ ability to connect people to remote mental health care, but also to applications of these technologies that make healthcare providers to deliver low cost, safe, and effective digital mental health care solutions to their patients.

On the basis of genetic information of each patient, the traditional machine learning approaches could accurately predict the treatment outcomes of multiple ketamine infusions (3). In fact, AI technologies in mental health, especially those based on deep learning algorithms, have already been applied to many areas (4, 5), including psychiatric diagnoses, symptom tracking, disease course prediction, and even psychotherapy (6). The emerging AI-based technologies include those conversational applications that teach the user emotional coping mechanisms and provide support for people with communication difficulties (7), computer generated audio‐visual character that forms the basis of avatar therapy (8), and intelligent psychiatrist-like robots with new advances in digital and AI technologies (9).

**Figure 1:**
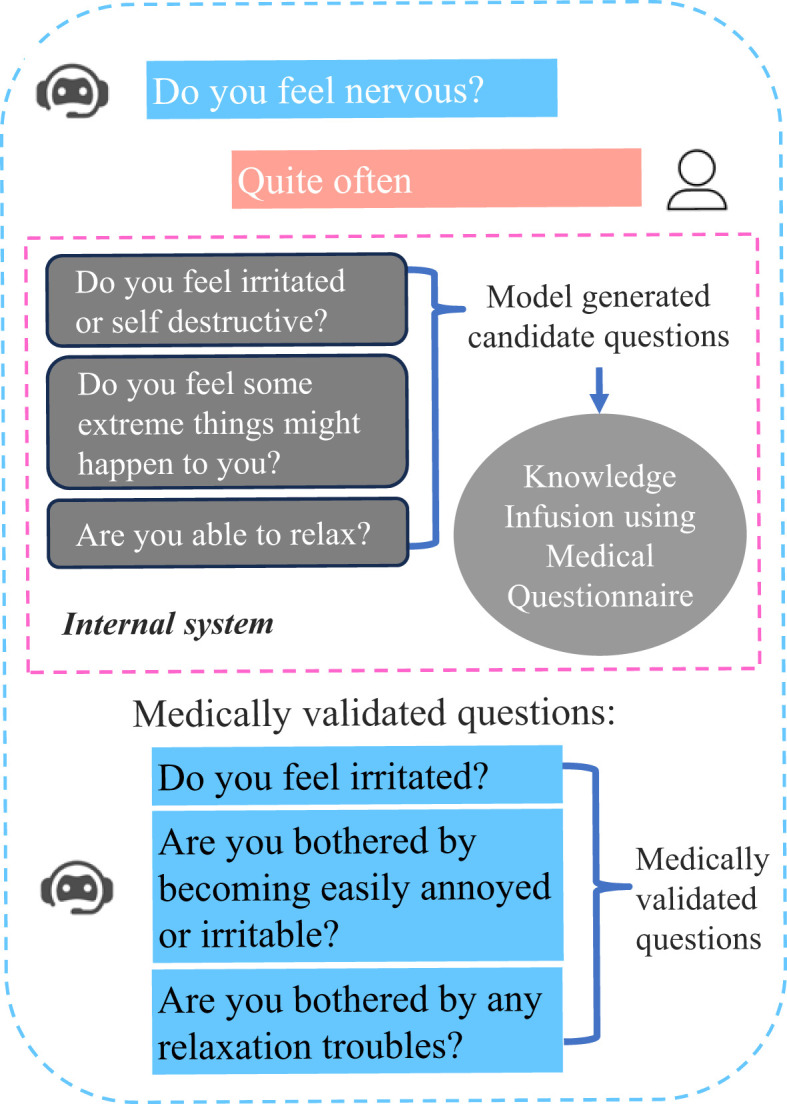
Illustration of questions generated by a conversational agent trained with ProKnow-algo. The blue part is generated by conversational agent, and the pink part is the answer of the user. The gray part represents the internal processing, and the red characters are not considered to be safe for mental patients.

Current virtual mental health assistants usually contain a chat feature, psychological assessment, an emotion detection module and a recommendation system for improving the mood of the user (10, 11). But they cannot be used for clinical scenarios because they do not strictly comply with safety constraints and specialized clinical processes. ProKnow (12) has proposed a method that the evidence-based guidelines and categories of conceptual understanding of experts in a domain were mapped to process knowledge. ProKnow-algo also has integrated the process knowledge by three aspects, that is, explicitly modeling safety, knowledge capture, and explainability (see **Figure 1**), and showed an averaged 82% improvement relative to other pre-trained large language models (LLMs) regarding to its three newly introduced evaluation metrics.

**Figure 2:**
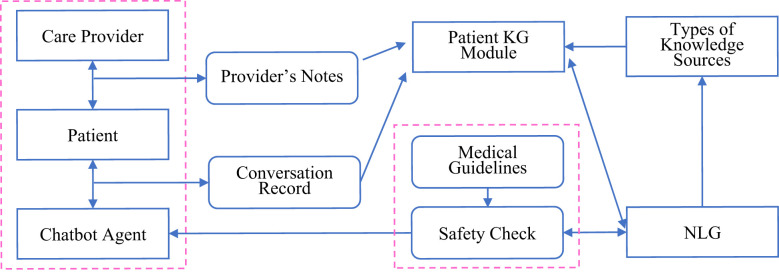
Formation of a personalized patient knowledge graph by using Alleviate to integrate the extracted knowledge with patient-specific information. Its task executions conform strictly to clinically established clinical standards and guidelines in the form of knowledge graph contexts. Its algorithms support constant feedback-based refinements through continued patient and care-provider interactions in a reinforcement learning mechanisms.

The implications of incorporating large-scale pre-trained models (PTMs) into the clinical psychiatric practices offer new perspectives on how AI-based chatbots would impact the delivery of mental healthcare (13). However, enabling practical capabilities in psychiatric agents raises several core practical issues on: (a) precise and individualized patient understandings beyond merely languages, (b) safety-constrained and clinically validated patient-agent interactions, and (c) feedback-based improving in real world settings, respectively. Addressing these practical issues is of paramount importance in the development of such AI-based chatbots. Alleviate (14) is a chatbot designed to provide personalized care for patients who suffer from mental health challenges, which complies strictly with the clinically established guidelines by leveraging the advantages of medical knowledge consolidated in the knowledge graph, and thus ensures effective and safe interactions with the patients (see **Figure 2**).

This review consists of four main sections by focusing on the AI technologies, available clinical evidence, and the implementation challenges of integrating digital and AI techniques into digital psychiatric systems. In the first section, we discuss the digital human technologies, LLMs and autonomous agents, psychiatric knowledge representations, and VR as the underlining techniques in the digital psychiatry revolution. In the second section, we introduce the clinical practices which guide our implementations of the framework. In the third section, we propose a blueprint of the framework towards digital psychiatry. In the last section, we discuss the challenges towards implementation and potential solutions for facilitating concrete application of digital psychiatry into real-world mental health care.

## 2 Current digital tools and technologies for psychiatry

To date, developments of current digital tools and technologies can be summarized from two different perspectives, i.e., a hardware perspective, and an AI perspective, respectively. From a hardware perspective, customer service robots, VR headsets as well as wearable devices are becoming more and more convenient to use, making them to be acceptable devices to reach for a majority of potential users. The rich and varied sensors equipped on VR devices can utilize heterogeneous data capturing and interactive/immersive power to deliver personalized monitoring and interventions.

**Figure 3:**
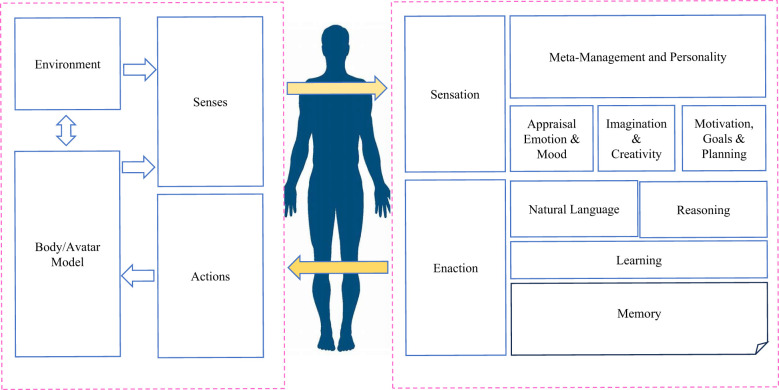
**A** typical technical illustration of a digital human. Besides the core architecture areas of a digital human, an important component of a digital human is how we can use knowledge graph and semantic triples techniques to encode what a person knows (semantic and procedural knowledge) and has experienced in the past (episodic knowledge).

From an AI perspective, large-scale PTMs have achieved notable successes, demonstrating emergent capabilities towards human-like intelligence (15, 16). Building upon these potencies, there is an increased interest that uses different kinds of PTMs as key controllers to construct autonomous agents with a goal of obtaining human-like decision-making capabilities. Under this research direction, numerous promising models have been proposed, where the main idea is to equip PTMs with mankind’s memory and planning capabilities to enable them behave as close as human beings do and accomplish a wide range of tasks effectively (**Figure 3**) (17).

According to the above discussions, AI-enabled devices have the great potential for improving mental health care to attain the following objectives: a) accumulating mental health data for daily and clinical usages; b) leveraging data and knowledge via large-scale pretrained models to generate actionable insights and predictions for clinical purposes; and c) providing a broad range of digital solutions without resorting to real-world clinical facilities. **Table 1** provides a detailed list of available digital tools and technologies which may be used for digital psychiatry. In the following sections, we explain these technologies and their potential applications in more details.

**Table 1:** Current digital tools and technologies available for digital psychiatry.

Technology	Tool	Year	Feature	Web Link
Digital Human	Hallo2	2024	Hallo2 was the first method to achieve 4K resolution and generated hour-long, audio-driven portrait image animations enhanced with textual prompts.	https://doi.org/10.48550/arXiv.2410.07718
	EchoMimicV2	2024	EchoMimicV2 leverages a novel Audio-Pose Dynamic Harmonization strategy, including Pose Sampling and Audio Diffusion, to enhance half-body details, facial and gestural expressiveness, and meanwhile reduce conditions redundancy.	https://doi.org/10.48550/arXiv.2411.10061
LLM	DeepSeek-R1	2025	DeepSeek-R1 achieves performance comparable to OpenAI-o1-1217 on reasoning tasks.	https://doi.org/10.48550/arXiv.2501.12948
	OpenAI o3	2024	OpenAI o3 is able to automate workflows, accelerate hypothesis generation, and foster interdisciplinary collaboration positions.	https://openai.com/index/introducing-o3-and-o4-mini/
	CBT-LLM	2024	CBT-LLM is a large-scale language model specifically designed for Cognitive Behavioral Therapy techniques. Empirical evaluations demonstrate that CBT-LLM excels in generating structured, professional, and highly relevant responses in psychological health support tasks.	https://doi.org/10.48550/arXiv.2403.16008
Autonomous Agent	Manus	2025	Manus is the world's first general AI Agent developed by Monica.im team, breaking through traditional AI's conversational interaction, achieving full-chain task processing from thinking to execution.	https://www.manusai.io/
	ClinicalAgent	2024	ClinicalAgent is a clinical multi-agent system designed for clinical trial tasks, leveraging GPT-4, multi-agent architectures, LEAST-TO-MOST, and ReAct reasoning technology.	https://doi.org/10.48550/arXiv.2404.14777
Knowledge Representation	KBLaM	2025	Knowledge Base-Augmented Language Model (KBLaM) is a novel approach that integrates structured knowledge bases into pre-trained LLMs.	https://doi.org/10.48550/arXiv.2410.10450
	GraphRAG	2024	The GraphRAG process involves extracting a knowledge graph out of raw text, building a community hierarchy, generating summaries for these communities, and then leveraging these structures when perform RAG-based tasks.	https://doi.org/10.48550/arXiv.2501.00309

AI, Artificial Intelligence; CBT, Cognitive Behavioral Therapy; GPT, Generative Pre-trained Transformer; LLM, Large Language Model; RAG, Retrieval-Augmented Generation.

### 2.1 Digital humans

One of the largest issue in healthcare is considered to be staffing shortages, which has been predicted to reach a crisis level in the coming years (https://www.who.int/health-topics/health-workforce). It has been predicted by the International Council of Nurses (ICN) that more than 13 million nurses worldwide are needed to fulfil the gap in the nursing shortage by 2030 (18).

In order to address the shortages of human staffing, digital humans (also referred to as virtual humans) provide us a viable practical solution. Digital humans refer to computer-generated entities that resemble human beings in appearance, behavior and communication abilities (19). They are designed to interact with humans and perform certain actions, using natural language processing, and other AI techniques. Digital humans can generate lifelike features, including facial expressions, gestures, and emotional responses, etc (20), which have been created as tools and artificial companions in video games, film production, human factors, and usability studies in a number of industries such as aerospace, automobile, clothing industry, and telecommunications.

In mental health scenarios, digital humans can encourage chronic care management or treatment adherence. They can also help patients to get familiar with processes and procedures, minimize stress, and reduce trepidation. As clinician burnout rises, digital humans are expected to play significant roles in alleviating current staffing shortages and challenges in delivering high-quality healthcare at all times and places. As the field of digital psychiatry continues to evolve, digital humans will hold immense potential power for more patient-centric and technologically advanced hospitals of the future.

A digital human generally consists of a combination of a number of technologies, which comprises two main components, i.e., body and mind, which are elaborated in the following.

1) The Body: The body is the embodiment of the digital human of the “real” world, in most cases as a 2 dimensional (2D) or 3 dimensional (3D) generated digital avatar (21). However, there is no reason for a digital human to be limited for only one representation in most scenarios (22). Speech is another key component among many other forms of representations, and it may leverage the generated audio data using voice cloning and generating techniques. Further, the dynamic interactions with speech, animation, gesture and expressions on specific topics are also required.

2) The Mind: The mind for a digital human is defined such that autonomous agents need to be capable of mimicking the mechanisms how human brain and human mind work. The major functional areas that the mind needs to possess include: the ability to sense and attend in both digital and “physical” senses, flexible conversational capabilities (including natural language recognition), the ability to generate a good understanding, the capacities of setting effective goals and performing efficient management tasks, the ability of keeping a wide spectrum of memories encompassing semantic, procedural and episodic information, the ability of maintaining a good emotion and mood, the ability to learn and reason, as well as the ability of having a long-term meta-management/meta-cognition functions. Taken together, body and mind are two indispensable parts of a digital human in digital psychiatry.

### 2.2 Large language models and autonomous agents

We would like to highlight several key examples in the development of LLMs and autonomous agents. First of all, in the year of 2018, OpenAI released its first LLM and Generative Pre-trained Transformer (GPT), subsequently, other LLMs were released by other developers like Google and Meta. Four years later, OpenAI released an updated LLM called ChatGPT, which quickly attracted world-wide attention due to its potent human like text generation and problem-solving capabilities. This was achieved through a novel technique, known as reinforcement learning from human feedback (RLHF), thus, generating much more reasonable and reliable outputs than previous LLMs (15).

Very recently, DeepSeek-R1-Zero trained with a large-scale reinforcement learning (RL) process without supervised fine-tuning (SFT) as a preliminary step, shows competitive reasoning capabilities. Furthermore, by incorporating multi-stage training and cold-start data before the RL process, DeepSeek-R1 achieves performance comparable to OpenAI-o1–1217 on reasoning tasks (23). ChatGPT O1 uses chain-of-thought reasoning (CoT) to enhance the structured problem-solving capabilities, while DeepSeek-R1 introduces self-reflection capabilities through reinforcement learning. The adoption of advanced reasoning models, such as ChatGPT O1 and DeepSeek-R1, represents an essential step forward in clinical decision support (24).

**Figure 4:**
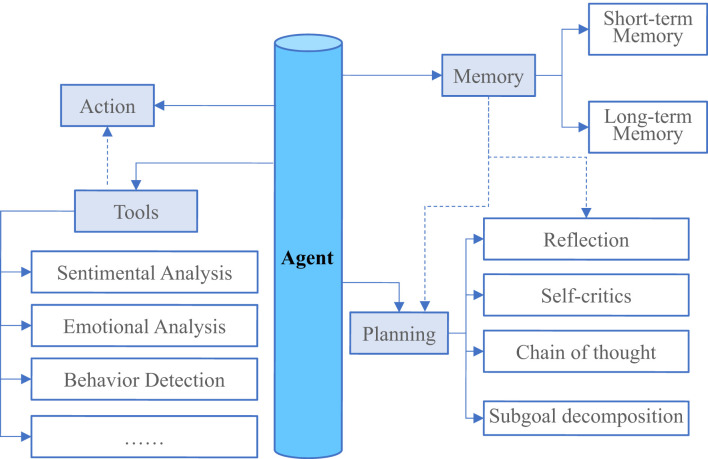
An overview of a large language system model (LLM)-powered autonomous agent system. In a typical LLM-powered autonomous agent system, LLM functions as the agent’s brain that is complemented by several key components like planning, memory, and tool use etc.

Besides LLMs, autonomous agents are recognized as an attractive technique to achieve artificial general intelligence (AGI) designed to accomplish various pre-defined tasks via self-directed planning and actions. Most agents developed in previous works are postulated that they can only learn in isolated and restricted environments on the basis of simple and heuristic policy functions (25–27). Such assumptions differ greatly from the human learning process as the latter has much more sophisticated mechanisms and individuals are able to learn from much wider environments. Thus, the agents developed from the previous studies can never replicate the human-level decision processes, especially in unconstrained and open-domain settings (17). LLM-based autonomous agents, therefore, promise to moderate these limitations by leveraging the emerging capabilities of LLMs (see **Figure 4**).

With the shortage of mental health workers coupled with the frequent in-person visits at clinics, LLM-powered autonomous agents offer a promising solution in helping patients to reduce their clinical symptoms during the early stages using autonomous services designed for timely prevention and intervention (28). Current chatbots are usually engaged in devising rule- or script-based screening or psychotherapy tasks to help patients manage their mental health by interacting with the chatbot applications during their daily uses. However, a practical autonomous agent’s capabilities are far from merely communications, since it also needs to accomplish specific tasks through interacting with the environments such that it can evolve itself like a real human. Therefore, to integrate autonomous agents with existing LLMs, an essential method is to assist LLMs in maximizing their capabilities by designing rational agent architectures. Recently, a Multi-disciplinary Collaboration (MC) framework has been proposed for the medical domain and it enhances the existing LLMs’ capabilities by leveraging the role-playing agents who involve in a collaborative multi-round discussion (29).

### 2.3 Psychiatric knowledge representation

The autonomous agents discussed above are often becoming fragile and can exhibit unsafe behavior during interactions with patients if omitting explicit clinical supervision from external knowledge sources. Psychiatrists often follow certain guidelines like Diagnostic Statistical Manual (DSM)-V, International Classification of Diseases (ICD)-10 and questionnaires like Hamilton Rating Scale for Depression (HAMD)-17 and Brief Psychiatric Rating Scale (BPRS) to gather firsthand patient mental health information (30). These guidelines and questionnaires provide the paradigms what we consider process knowledge. Incorporating process knowledge as additional components of the autonomous agents can guide the LLMs to capture information relevant to clinical practices and avoid the conversational systems to steer the topics of conversation into the contents which are not chartered (31).

**Figure 5:**
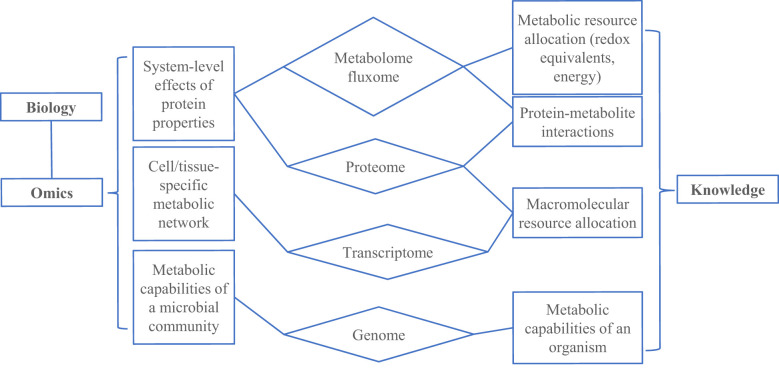
An overview of synthesizing knowledge using omic data and genome-scale models (GEMs). GEMs convert the reactions (related to metabolism, transcription and translation) that occur in an organism to a mathematical formulation that can be modeled using optimization principles.

Analysis of multi-omics data offers powerful tools to reveal the disease mechanisms and to assist the findings of molecular biomarkers (32, 33). Epigenome-wide association analysis points to various gene-regulatory mechanisms and environmentally-induced post-translational modifications that account for mechanistic alterations and biological heterogeneity in many psychiatric disorders (34). Transcriptomics analysis explores the broad set of RNA transcripts which can be used to map out clinically relevant gene expression signatures of different neurological and psychiatric disorders (35). Researchers also use proteomics to identify the ultimate pathophysiological mechanisms as well as to develop, validate, and qualify bodily fluid biomarkers in psychiatric diseases like Alzheimer’s disease (AD), Parkinson’s disease (PD), and schizophrenia (36). Besides, metabolomics and lipidomics are expected to provide individualized information about bioenergetic, metabolic, and lipid homeostasis processes, relevant to critical pathophysiological pathways that occur in neurological and psychiatric diseases (37). Multi-omics technologies hold the potential to completely read out the molecular state of a cell at different biological scales. Thus, the integration of multi-omics data can provide a comprehensive view of the entire biological system. So far, various genome-scale modeling methods have been used to interpret multi-omics data, which include genomics, transcriptomics, proteomics, metabolomics, and meta-omics (**Figure 5**). Therefore, it is important for mental health systems to synthesize such diverse biomedical knowledge through the integration of multi-omics data with appropriate models (38–40).

Reported studies have identified various structural and functional endophenotypes for cognition, behavior, and movement, and also related alterations in neurological and psychiatric disorders (41). Many researches have revealed that the resting state functional connectome of the brain has shown promise in differentiating individuals with specific neurodevelopmental conditions, e.g., autism spectrum disorder (ASD) from typically developing controls (42) and predicting an individual’s response to treatment in anxiety (43) or depression (41). Changes in regional and whole-brain functional architectures on the millisecond time scale can reflect physiology- or disease-related alterations in the brain (44, 45). In view of the low temporal resolution of magnetic resonance imaging (MRI), this methodology can be complemented by electroencephalography (EEG) and magnetoencephalography (MEG) both offering indirect and noninvasive assessments of neuronal activity at high temporal resolution. Recent researches have utilized the integrated data from genomic and EEG technologies to find those genomic variants affecting brain synchrony, and offer new mechanistic insights into genetic variants associated with alcohol use disorders and epilepsy (46). Moreover, bioimaging data implemented in Product Life Cycle Management (PLM) have been used to solve their evolution problems (47). Taken together, omics and bioimaging data not only facilitate and boost the building of knowledge about genetics, risk factors, and molecular pathways relevant to various psychiatric diseases, but also can be used as knowledge to aid the predictions and decisions during the clinical practices.

Besides, most existing medical AI models typically lack prior knowledge of the clinical, biological and medical domain before they are finetuned for their downstream tasks. Instead, they rely mostly on the learned statistical associations between features of the input data and their targets, without having contextual information such as diversified clinical factors. This makes it even harder to train or finetune models for specific clinical tasks, particularly the clinical data are hard to acquire in most cases. To some extent, generalist medical AI (GMAI) models try to mitigate the above limitations by formally integrating different kinds of knowledges. The structures such as knowledge graphs can allow GMAI models to identify biomedical concepts and their relationships by reasoning mechanisms of GMAI. Furthermore, GMAI can retrieve relevant context from existing databases, in the form of articles, images or entire previous personal clinical records by leveraging novel retrieval-based approaches (48, 49). A GMAI model may solve tasks with limited data by utilizing knowledge of related problems such as AI-based drug repurposing (50). Furthermore, GMAI offers opportunities accomplish a nearly unlimited range of medical tasks by interchangeably parsing multiple data modalities, learning new tasks, and leveraging domain knowledge. The flexibility of GMAI allows models to stay robust in new settings and keep track of updated knowledge and technologies without needing to be constantly retrained from scratch.

### 2.4 Virtual reality and psychology

VR often provides an immersion experience in an interactive and simulated environment by wearing a headset. Its ability to mimic exposure to real­world environments presents important opportunities for not only mental health assessment but also treatment (51). Currently used mental health assessments are limited by a lack of clinical validity and relying mostly on subjective judgements (52). VR can be used to monitor patients’ responses to stimuli within controlled virtual environments, and hence provides pivotal insights into the way in which clinically relevant feedback develop in real world (51, 53). Compared to the real-world cases, controlled exposure to anxiety­inducing stimuli within a virtual environment often offers a much more efficient way to deliver exposure­based behavioral treatments. The VR treatment has many advantages such as the repeated exposure to feared stimuli, enabling the individual to trigger, and healthy responses in a safe and controlled therapeutic platform (54, 55).

A recent meta­review focused on anxiety disorders and post-traumatic stress disorder (PTSD), revealed that effect sizes of VR exposure treatments were ranged from moderate to large and were even maintained at follow­ups (56). A small number of clinical trials have been also conducted for other psychiatric diseases, and increasing evidences showed that VR treatments might be effective for depression (57), schizophrenia (56) and eating disorders (58). Besides, a few studies have explored VR treatments beyond the abovementioned exposure therapy, which have yielded positive results (59). Further, several pilot studies have also shown that patients can even learn therapeutic skills such as mindfulness (60), relaxation (61) and self-compassion (62) with carefully designed VR systems. In the future, the immersion experience of VR may help people develop skills to manage mental health difficulties with increased treatment engagement and efficacy.

Virtual worlds created by VR technologies can also enable users to immerse themselves within virtual environments, represent as personalized avatars, and interact with other users in real-time. By this way, it offers a compelling solution to increased demand for technological platforms that can deliver personal clinical care digitally (63). Although, few studies have been conducted in psychiatric care, there have been promising results especially in psychosis (64). Digital therapy including VR treatment has the potential of offering highly accessible care within personally tailored, engaging therapeutic environments that provide a low-cost, safe and comfortable medium for mental health solutions.

## 3 Clinical topics for psychiatry that can be addressed by a digital paradigm

**Figure 6:**
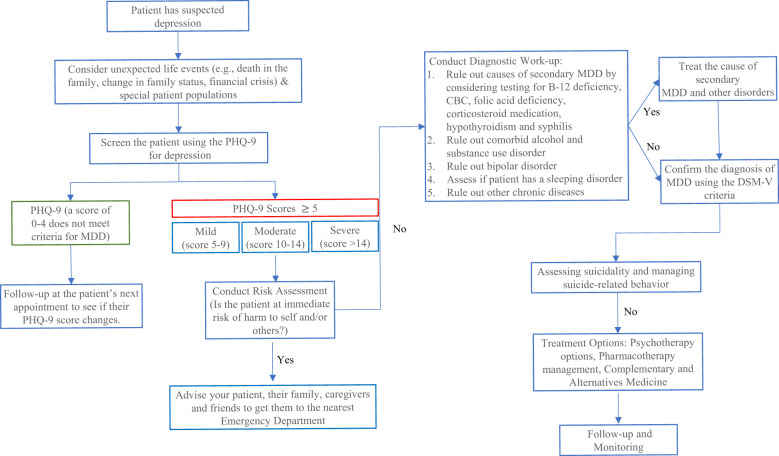
An example of adult Major Depressive Disorder (MDD) diagnosis pathway. Every clinical encounter with a MDD patient should include an assessment of suicide risk. To assess if a patient is at risk of suicide or developing suicidal thoughts by using the Columbia-Suicide Severity Rating Scale (C-SSRS). Depressed Mood (Q1) and/or loss of interest/pleasure (Q2) are also required to be present. It is also important to provide the patient with non-judgmental care (e.g., Being diagnosed with depression is nothing to be ashamed of, it is very common and many adults are diagnosed with it every year).

Before going to our proposed framework for digital psychiatry, we first introduce some fundamental practices used in most of the current psychiatric clinics. Please also refer to **Figure 6** for the typical clinical diagnosis and treatment processes of adult major depressive disorder (MDD). These practices or processes could be fully automated by using the digital psychiatry methodologies.

### 3.1 Clinical signs and symptoms

A mental health concern becomes a mental disorder when those ongoing clinical signs and symptoms cause frequent stresses and affect the person’s normal abilities to function. In most cases, these clinical symptoms can be mitigated by a combination of medications and psychotherapy. Unfortunately, the signs and symptoms of mental illness can vary significantly, depending on the types of disorders, circumstances and many other factors. This is critical while designing AI systems for psychiatry. It is known that mental illness symptoms also affect emotions, thoughts, and even behaviors. Thus, we should acquire and track most of the signs and symptoms using appropriate questionnaires and multi-modal techniques conducted by immersive conversational agents.

It is also important to note that symptoms of a psychiatric disorder can appear as physical problems, such as stomach pain, back pain, headaches, or other unexplained aches and pains. Thus, the physical signs and symptoms should be considered while designing the digital psychiatric systems.

### 3.2 Cause analysis and risk factors

Generally, psychiatric disorders are thought to be caused by various biomedical and environmental factors as summarized below. 1) Inherited traits: Mental disorders are generally more common in people whose biological relatives also have a mental illness. Certain genes can increase an individual’s risk of developing a mental illness, which can also be triggered by the life situation faced by the person of interest. 2) Environmental exposures before birth: Exposure to environmental stressors, inflammatory conditions, toxins, alcohol or drugs while in the womb has been linked to a mental illness. And 3) Brain chemistry: Neurotransmitters are naturally occurring brain chemicals that carry signals to other parts of brain and body. When the neural networks involving these neurotransmitters are impaired, it would change their functions, leading to depression and other mental disorders.

Moreover, there are also some other factors that may increase the risk of causing a psychiatric disorder, which include but not limited to: a direct family member with a history of mental illness; living in a stressful life situation (e.g., financial hardships, loss of a loved one or a divorce); an ongoing medical condition (e.g., a physical injury, or traumatic experiences like military combats); use of psychoactive drugs (e.g., alcohol, marijuana, and others); a childhood history of abuse or neglect; having few friends or few healthy interpersonal relationships; and a previous diagnosed mental illness (e.g., schizophrenia) etc.

The symptoms of mental illnesses can be a recurrence, a relapse, or a long-lasting duration of a pre-existing mental disorder. A potential patient might have multiple mental health disorders at a given period of time. Moreover, mental illnesses sometimes could be complicated by a wide range of factors that include: a lack of happiness and a decreased enjoyment of life, a family-related life event, a deficiency of social skills to interact with peers (e.g., in school or at workplace), usages of tobacco, alcohol and other abused drugs, legal or financial problems, poverty, either self-harm or harm to others, a weakened immune system such that the physical body has a difficult time in resisting either acute or chronic infections, cardiovascular diseases and other medical conditions.

Regarding to the above situations, we could compile the above factors collected from clinical and research scenarios into the knowledge-based PTMs such as GMAI to analyze the causes of an individual’s mental illness. Thus, it is important to identify, define and build the relations between these factors and a mental illness during the knowledge construction.

### 3.3 Diagnosis

Obtaining an accurate diagnosis constitutes the first step first in helping improving the health of a patient with mental illness. To conduct an appropriate diagnosis and to check for related medical and physical conditions, a patient may need to undertake: 1) a physical exam to identify any physical problems that could cause that patient’s mental symptoms, 2) clinical laboratory tests which may include a check of patient’s thyroid function, a screening for alcohol and other psychoactive drugs, as well as 3) a comprehensive psychological evaluation by a trained mental health professional through talking to a patient about his/her symptoms, thoughts, feelings and behavior patterns. In addition, patients sometimes may also be asked to fill out questionnaires to conform a diagnosis.

In many cases, it is difficult to determine which mental disorder might cause a patient’s symptoms. The more information a patient provides, the more the medical staff will understand what patients’ symptoms may represent. The results of a physical exam and laboratory tests can be interpreted by the knowledge-based PTMs such as GMAI or LLMs. On the other hand, DSM-V or clinical questionnaires conducted in the psychological evaluation process can be considered as process knowledge for steering the natural language generation (NLG) models to recognize information relevant to diagnosis and the topics of conversation.

### 3.4 Treatment

After making a proper diagnosis, there is no question that an effective treatment plan should be developed that primarily depends on the type and severity of a diagnosed mental illness, and acquiring essential information on previous treatment history of the patient is also important. In many cases, a combination of different treatment methods might be the best option. If a patient has a mild mental illness with well-controlled symptoms, treatments conducted by a primary care provider may be sufficient. However, sometimes it is more appropriate and effective to have a team approach such that all psychiatric, medical and social needs of a patient are considered. This is especially true for severe mental illnesses, such as schizophrenia.

We just list the most commonly used classes of prescription psychiatric medications as follows: 1) Antidepressants: They are used to treat depression, anxiety and sometimes other conditions by improving symptoms such as sadness, hopelessness, lack of energy, difficulty concentrating and lack of interest in activities. 2) Anti-anxiety medications: These drugs are generally used to treat anxiety disorders, such as generalized anxiety disorder or panic disorder by reducing agitation and insomnia. 3) Mood-stabilizing medications: These mood stabilizers are most commonly used to treat bipolar disorders, which involves alternating episodes of mania and depression. And 4) Antipsychotic medications: They are typically used to treat psychotic disorders, such as schizophrenia. In most cases, psychiatric medications often could not cure a mental illness, but they can often greatly improve symptoms. The most effective medicine treatments for a patient would depend on patient’s particular situation and how a patient’s body responds to them. Thus, it is critical to include the holistic information and possible interactions between a medication and a mental illness into the knowledge-based PTMs.

Psychotherapies represent important regimens in treatments of patients with mental illnesses. These psychotherapies often involve talking about a patient’s condition and related issues with a clinician. By this way, patients can learn about their mental conditions such as moods, feelings, thoughts and behavior with psychotherapy. As psychotherapy such as cognitive behavioral therapy (CBT) is designed to be a template-based therapy, professionals can scrutinize patients by checking their behavior following the psychotherapy rules. If there is a conversational AI agent available, there would be no need for a clinician to complete this task personally. However, to provide clinical supports for mental illnesses, an AI system would require a validation among the patient’s responses, medical knowledge and the clinician’s expertise. This is also required to ensure safe and effective conversations between the patient and a conversational agent.

## 4 Proposed framework for digital psychiatry

**Figure 7:**
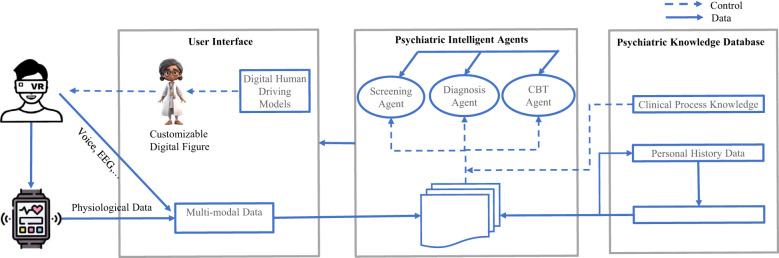
An overview of the digital psychiatric framework. We divide the framework into three main parts: the user interface interacts with patients with digital devices and retrieving multi-modal data from these devices at the same time; the Psychiatric Intelligent Agents control and coordinate the agents powered by large-scale pretrained models with different functions; the Psychiatric Knowledge Database guides all the agents with crafted psychiatric knowledge in different domains.

To meet the pressing needs faced by psychiatry professionals, we propose an integrated framework of digital psychiatry that consists of three main parts: 1) usage of immersive user interfaces using digital human and VR technologies, 2) application of psychiatric autonomous agents based on large-scale PTMs, and 3) construction and application of effective clinical/biomedical knowledge databases related to psychiatry. Such proposed framework should integrate “real-world” inputs and AI-generated user interfaces together such that the system can strictly conform to clinically validated processes/criteria via knowledge supported modules, intelligent agents based on behavior and physiological inputs from different types of devices and clinical scenarios (**Figure 7**). Please refer to **Table 2** for the current researches on frameworks for digital psychiatry.

**Table 2:** Current researches on frameworks for digital psychiatry.

Framework	Used Tools	Year	Feature	Web Link
Agent Hospital	LLM, autonomous agent	2024	Agent Hospital simulates the entire process of treating illness. All patients, nurses, and doctors are autonomous agents powered by LLMs.	https://doi.org/10.48550/arXiv.2405.02957
PSYCHE	LLM, conversational agents	2025	PSYCHE is a novel framework designed to enable the clinically relevant, ethically safe, cost-efficient, and quantitative evaluation of psychiatric assessment conversational agents.	https://doi.org/10.48550/arXiv.2501.01594
EmoAgent	LLM, Character-based Agent	2025	EmoAgent is a multi-agent AI framework designed to evaluate and mitigate mental health hazards in human-AI interactions.	https://doi.org/10.48550/arXiv.2504.09689
ChatMDD	digital human, autonomous agent & knowledge graph	2024	ChatMDD aims to automate the entire process of treating mental illness. All the interfaces and processes are autonomous agents powered by LLMs and psychiatric knowledge graphs.	https://doi.org/10.3389/fpsyt.2024.1417253

AI, Artificial Intelligence; LLM, Large Language Model; LLM, Large Language Model; MDD, Major Depressive Disorder; VR, Virtual Reality.

### 4.1 Virtual reality-enabled conversational user interface

To address fundamental interaction challenges for immersive systems, one needs to build novel user interface concepts by utilizing a combined approach of rapid prototyping and experimentation with rigorous empirical studies of human perception and spatial cognition following the psychiatric guidelines. VR provides a highly immersive experience as the player’s actions interact with the virtual world in real time, and feedback is immediately reflected. VR immersive technology allows users to experience enhanced reality using human–computer interfaces (HCI). Many systems have implemented VR with improved HCI to provide competitive products for many different kind of applications (65, 66).

With the rapid advancement of the field of AI, we can truly leverage the power of the VR devices and digital human technologies to create VR agents that have a “human-like” feel to them. These agents, empowered by digital human, which could look, talk, provide hand gestures and facial expressions like real clinical staff, can be driven by autonomous agents via generated texts, voices or videos (67). Each user can choose the figure and voice he or she feels the most comfortable to chat with (68, 69), which has a natural way of talking and a body language that is easy to read, anticipate and interact with. In particular, it is noteworthy that human-like avatar can provide advices, make a diagnosis, psychotherapy, and is always ready to provide services (70).

### 4.2 The psychiatric agents

Multi-Agent Systems (MAS) (71) and Multi-Agent Collaboration (MAC) (72) are means through which agents could solve complex real-world problems. They implemented the communication and cooperation mechanisms between agents, which are commonly considered as autonomous units to solve intractable tasks. MAS has been applied in many practical applications such as robotics and medical applications (71). Besides, MAS and information technology could also be combined to improve the palliative care provided to the patients (71).

As illustrated in **Figure 7**, in our integrated framework, we aim to leverage these above techniques to build the psychiatric agent layer. The components of the psychiatric agent layer are composed of a set of psychiatric agents (such as agent for safe check, agent for diagnosis, agent for psychotherapy, … etc.), and a collection of PTMs controlling and coordinating these agents. Each agent may be supported by the domain knowledge necessary to control or guide the agent; hence, our agent components combined could provide a practical means of addressing diverse clinical challenges. The PTMs also serve as the environment variables allowing the agents to adapt to incoming information in time. Due to the communication mechanisms between agents, any agent can provide and ask for services. These agents may proactively warn or notify the user, even when the user has not realized those risks being executed. The essential property of any agent is its autonomy in most of the situations. Agents may also exhibit goal-directed behaviors by adding properties for finding relevant information and show it before the user requests it. Autonomous agents are required to process individual goals and self-resources without any direct external intervention. That is, they have some degree of control over its actions and internal state and regulates its functions without outside assistance or supervision.

### 4.3 Knowledge-based databases

Except for the knowledge discussed in Section 2, it is also important to collect and monitor the physiological and functional data for the digital agents to work perfectly. Neurological and psychiatric diseases are often manifested in several physiological systems and functional domains such as changes in complex behaviors, social interactions, and sleep patterns. AI enabled data collection can often capture more effective repertoire of disease-related phenotypes. Most digital health technologies hold the advantage of being portable, sustained, quantitative, and allowing data collection to be convenient, unobtrusive, and longitudinal. Digital devices can accumulate a multitude of health data such as heart rate, body temperature, cardiac rhythms, skin conductance, blood oxygenation, and cover clinically relevant behaviors such as motion, gait, pace, sleep, speech and voice patterns. By encoding the above time-aware health data with AI models, we can identify subtle changes during early stages of disease, and thus offer solutions for screening and early diagnosis. This also open new possibilities for collecting longitudinal data and have the potential to provide useful insights on prognosis and disease progression needed by the diagnosis agent.

### 4.4 Implementation recommendations

Based on our proposed framework, we think that a digital psychiatric clinic should include the following components (**Figure 7**). First, we built a 3D digital human interface using the voice cloning and audio-driven avatar video generation technologies. This interface is equipped with a VR headset for immersive interactions, which aims to enhance the engagement of the participator. The VR interface is driven by three autonomous AI agents which are empowered by three different types of LLMs for automating the screening, diagnosis, and CBT treatment processes. The screening LLM finetuned from the reasoning LLM model is used for scoring the questionnaires based on the transcription texts from the participator. The diagnosis LLM finetuned from the knowledge enabled multi-modal reasoning model is mainly used for diagnosing the types and the severity of the mental disorders. The CBT LLM is finetuned from the normal chat LLM and guided by the CBT goals for each session. By designing this way, we can not only automate the clinical processes, but also restore the clinical scenarios at maximum which are critical for mental patients. Besides, mobile digital health technologies can be used to track and monitor the mental states of patients. When alerts occur, the patient can use the digital psychiatric clinic devices to get assessment and treatment services remotely. As digital and intelligent technologies continue to improve healthcare delivery, make operational efficiencies, and enhance patient experience, they would change the mental health services with higher quality care, improved operational efficiencies, and increased patient satisfaction.

The evaluation of the digital psychiatry systems is challenging. We can divide the evaluation process into three levels. In the first level, we should evaluate each model used in the system one by one. In this level, we could leverage the existing evaluation methods and the evaluation data could be collected from the same or similar clinical tasks or generated from other powerful LLMs by the prompt engineering techniques. In the second level, we should evaluate each agent composed of the system one by one. In the last level, we should evaluate the whole system in the practical settings. In the last two levels, it is difficult to acquire enough evaluation data using the existing datasets or data generators. Thus, the evaluation process should be completed by certified clinicians in the standard clinical trial settings.

However, the main challenges of implementing digital psychiatry systems are not only the technologies or innovations themselves, but also the challenges related to instructing all the participants (i.e., patients/clinicians) and the context of mental health care delivery (e.g., regulation). Therefore, the most immediate benefits in the field can be realized by making the most of existing technologies in a real­world setting. While it is unlikely that there would be a single solution to these implementation challenges, various options are also available, regarding to local conditions and current clinical situations.

We explain several essential functions that our proposed framework should include: 1) Safe and explainable interaction processes: We should integrate the patients' mental states and physiological information with the clinical process standards together, i.e., to gain a comprehensive understanding of the patients' mental illnesses. The automation processes should be clear and transparent at any critical checkpoint and should handle the extreme or emergent cases at any time. Therefore, acquiring crafted process knowledge could be more important than just development of powerful models. The integrated framework should also have a critical component of safety check in order to detect potential conversation patterns that require emergency human intervention. 2) Knowledge based multi-modal PTMs: Powerful large-scale PTMs are often not enough in medical domains. Especially in mental health situations, trained clinicians use manuals and medical knowledge to make their decisions. These considerations cannot be ignored when designing autonomous psychiatric systems. Normally, we could utilize the retrieval-augmented generation (RAG) (73) or GrpahRAG (74) to enhance the PTMs. 3) MAS mechanisms: Adhering to the workflow of clinical practices, we should adopt the MAS infrastructure to ensure the psychiatric agents work properly. Because, this could enhance the reliability and capability of autonomous intelligent systems. Notably, LLM-based MAS are considered a promising pathway towards realizing AGI which is equivalent to or even surpasses the human-level intelligence (75). 4) Devices and user interfaces: The mental health patients can be easily affected by environment factors, such as the speech or appearance of clinicians. So, carefully designed devices and interfaces are also important (76, 77)

We also put forward the following recommendations around high-priority considerations for the practical implementations of digital psychiatry: 1) Privacy and security: Without a guarantee on privacy and protecting users’ medical and clinical data, digital psychiatry systems would lack the trust necessary for clinical acceptance. Across all conditions and technologies, ensuring privacy of patients’ data remains to be of the first importance, especially in mental health domains. 2) Efficacy: There is increasing evidence showing that the digital psychiatry is quickly becoming feasible and acceptable to those with mental health problems. As digital psychiatry seeks reimbursement or addition into national formularies, the need for high­quality data cannot be ignored any further. 3) Engagement: Available data suggest that engagement remains difficult for some mental disorder patients. Carefully designed immersive environments offer a feasible solution to sustain engagement. It is also necessary to conduct research on some issues like why people use digital psychiatry and how best to encourage sufficient engagement. And 4) Clinical integration: Although many efforts have been made, integration of digital psychiatry into clinical practice remains cumbersome. Therefore, leveraging current clinical services and re-designing medical care models are also required to fully realize the benefit of digital psychiatry. As digital health standards, policies and regulation are becoming more mature and sophisticated in the near future, the frameworks must also be prepared to offer viable solutions. A great understanding of the future potentials, key issues, and priority actions is most prospective in the light of the above discussions of challenges concerning stakeholders and clinical contexts.

## 5 Discussions and challenges

The recent advancements in the field of AI have brought dramatic influences in various medical arenas, and digital psychiatry represents an emerging novel approach in applying AI technologies and methods to overcome practical hurdles in psychiatry. To ensure all potential features of digital psychiatry are realized in real-world settings, all of the above-mentioned advances in both the technologies themselves and the researches supporting these advances should be considered and included if possible. Furthermore, a number of obstacles and dilemmas surrounding the implementation of digital psychiatry in clinical practices must also be resolved. At the patient level, ensuring user engagement with immersive devices, and how this is related to observed benefits, is greatly needed. At the provider level, implementation of digital psychiatry systems with required clinical facilities, clearer expectations of where the system should sit within the clinical workflow, and appropriate integration of the new system with existing ones is essential if the integration is to be at all possible. At the administration level, further legal measure is required to ensure that clinical regulations of digital psychiatry are flexible such that innovation and new technology advances can be effectively adopted within healthcare services, while stricter regulations for commercial settings are also equally important. Future AGI capabilities in empathy, emotion recognition, personality assessment, and detection of mental health warning signs are still needed to be enhanced in order for digital psychiatry to be effectively integrated into current psychiatric care.

Even now the performance of AI models is relatively strong, clinical acceptance of digital psychiatry is still far from guaranteed. For example, assistant-level solutions that flag potential drug interactions are typically easier to accept particularly if the autonomous systems disrupt existing workflows or raise concerns about loss of physician autonomy. Moreover, clinical staffs also need to be trained to appropriately interpret AI outputs and utilize the system’s recommendations.

Each of these implementation-related issues must be considered and resolved with a realization of the ethical issues which could run through the whole digital psychiatry process. Potential bias in training data or knowledge database is another critical concern. For example, if some demographic or geographic groups are underrepresented in historical medical records, the knowledge-based models might systematically overlook those populations. Further, the lack of reasoning can hamper trust if physicians could not understand how a recommendation is generated. However, the adoption of emergent AI reasoning models (e.g., DeepSeek-R1 and OpenAI o3) may mitigate this challenge. Data privacy requirements such as Health Insurance Portability and Accountability Act (HIPAA) in the United States or General Data Protection Regulation (GDPR) in Europe are also critical and essential for implementing the digital psychiatry framework, which often ingest sensitive personal information in real time. This not only affects where and how data are stored but also shapes the algorithmic design, e.g., requiring homomorphic encryption or differential privacy techniques to ensure secure processing. Currently, digital psychiatry is in its infancy, and a variety of crucial factors should be taken into consideration in its further growth and development.
